# Dissipation Behavior of Three Fungicides during the Industrial Processing of *Paeoniae* *Radix* Alba and Associated Processing Factors

**DOI:** 10.3390/ijerph16122196

**Published:** 2019-06-21

**Authors:** Sheng-Nan Li, Ming-Na Sun, Fan Wang, Xing Xu, Xin-Hong Zhang, Jin-Juan Ma, Jin-Jing Xiao, Min Liao, Hai-Qun Cao

**Affiliations:** 1 School of Plant Protection, Anhui Agricultural University, Hefei 230036, China; 13210615585@163.com (S.-N.L.); fxhlrh@163.com (F.W.); zxh1060559211@163.com (X.-H.Z.); majinjuan1024@163.com (J.-J.M.); xiaojj187012@163.com (J.-J.X.); liaomin3119@126.com (M.L.); 2 Institute of Plant Protection and Agro-Product Safety, Anhui Academy of Agricultural Sciences, Key Laboratory of Agro-Product Safety Risk Evaluation (Hefei), Ministry of Agriculture, Hefei 230031, China; sunmingna@126.com; 3 Provincial Key Laboratory for Agri-Food Safety, Anhui Agricultural University, Hefei 230036, China; xu99xing@163.com

**Keywords:** processing factors, fungicide, pesticides residues, *Paeoniae Radix* Alba

## Abstract

Before being administered as medicinal products, Chinese herbal medicines (CHMs) must be processed and decocted for human consumption. While the presence of pesticide residues in CHMs is a major concern, pesticide dissipation behavior during CHM processing has rarely been reported. In this study, the dissipation of three pesticide residues in the CHM *Paeoniae Radix* Alba (PRA) was investigated during each step of industrial processing. The boiling process was found to significantly reduce pesticide residues (61–89%), and the peeling process also contributed to pesticide degradation (29–68%). The high temperature (60 °C) during the drying process led to further pesticide degradation. The processing factors of all three pesticides after each processing step were less than one, and the processing factors for the overall process were lower than 0.027, indicating that industrial processing clearly reduced the amount of pesticide residues (97.3–99.4%). The findings provide guidance for the safe use of fungicides in CHMs and can help establish maximum residue limits for PRA to reduce human exposure to pesticides.

## 1. Introduction

Chinese herbal medicines (CHMs) are an important component of eastern and alternate medicine [1]. As Chinese medicine becomes more popular worldwide, the demand for CHMs and medicinal plants on the international market is growing [2]. However, the increased demand for CHMs has resulted in the commercialization of CHM production. As a result, the presence of pesticide residues in CHMs has become a significant safety concern [3,4]. Pesticide residues in herbal products may accumulate as a result of agricultural practices [5] including spraying, soil treatment, cultivation in contaminated soil, the use of contaminated water sources, and the administration of fumigants during storage. The agricultural production of herbal plants is followed by processing into prescription CHMs, with typical processing steps including boiling, steaming, adding salt or vinegar, frying, and burning [6]. Studies have shown that common processing steps, such as washing, peeling, blanching, juicing, fermenting, and distilling, can reduce the levels of pesticide residues present in foods [7]. For example, Shakoori et al. [8] studied the effect of the cooking method on the residues of 41 pesticides in rice and found that volatilization, hydrolysis, and thermal degradation decreased pesticide residues by 20.7% to 100%. Xiao et al. [9] studied the digestion behavior of five pyrethroids in mushrooms during processing and demonstrated that the pyrethroids had low processing factors (PFs) of 0.08% to 0.13%, indicating that the pyrethroid exposure risk of mushroom consumption was negligible.

PFs are defined as the residue level in the processed product divided by the residue level in the raw product. Investigations of PFs play an important role in assessing dietary exposure to pesticide residues via the consumption of processed products [10]. PF values greater than one indicate an increase in the concentration of pesticide residues during processing, whereas PF values less than one indicate a decrease. The PF value depends on both the crop type and the physicochemical properties of the pesticide, particularly water solubility and the water–octanol partition coefficient [11].

*Paeoniae Radix* Alba (PRA) is a common CHM that has been shown to have sedative, analgesic, anti-inflammatory, liver-protecting, and immune-regulating effects along with the ability to inhibit platelet aggregation [12]. As reported in the Chinese Pharmacopoeia, PRA is produced from the peony root that has been peeled and boiled. During industrial production, PRA must be boiled, cooled, peeled, dried, moistened, sliced, and dried a second time before finally becoming a medicinal product [13]. To clarify the digestion process of pesticides during PRA processing, three fungicides commonly applied during the growth of peony root used to produce PRA were selected as experimental agents. Pesticide-soaked PRA was processed using the modern industrial processing method, and samples were collected at each processing step to evaluate the pesticide residues using Quick, Easy, Cheap, Effective, Rugged, and Safe (QuEChERS)-ultra-performance liquid chromatography (UPLC)-tandem mass spectrometry (MS/MS) [14].

## 2. Materials and Methods

### 2.1. Chemicals and Reagents

Three pesticide standards (azoxystrobin, epoxiconazole, and difenoconazole; purity ≥98%) were purchased from Innochem Science and Technology Co., Ltd. (Beijing, China). Methanol and acetonitrile (liquid chromatography grade) were purchased from Tedia Company, Inc. (Ohio, USA). Anhydrous magnesium sulfate, primary-secondary amine (PSA), C18, and anhydrous sodium acetate were provided by Agilent Technology (USA). Stock standard solutions of the three pesticides were prepared in methanol and stored at −20 °C in a freezer. 

### 2.2. In-vitro Sample Preparation

**Figure 1:**
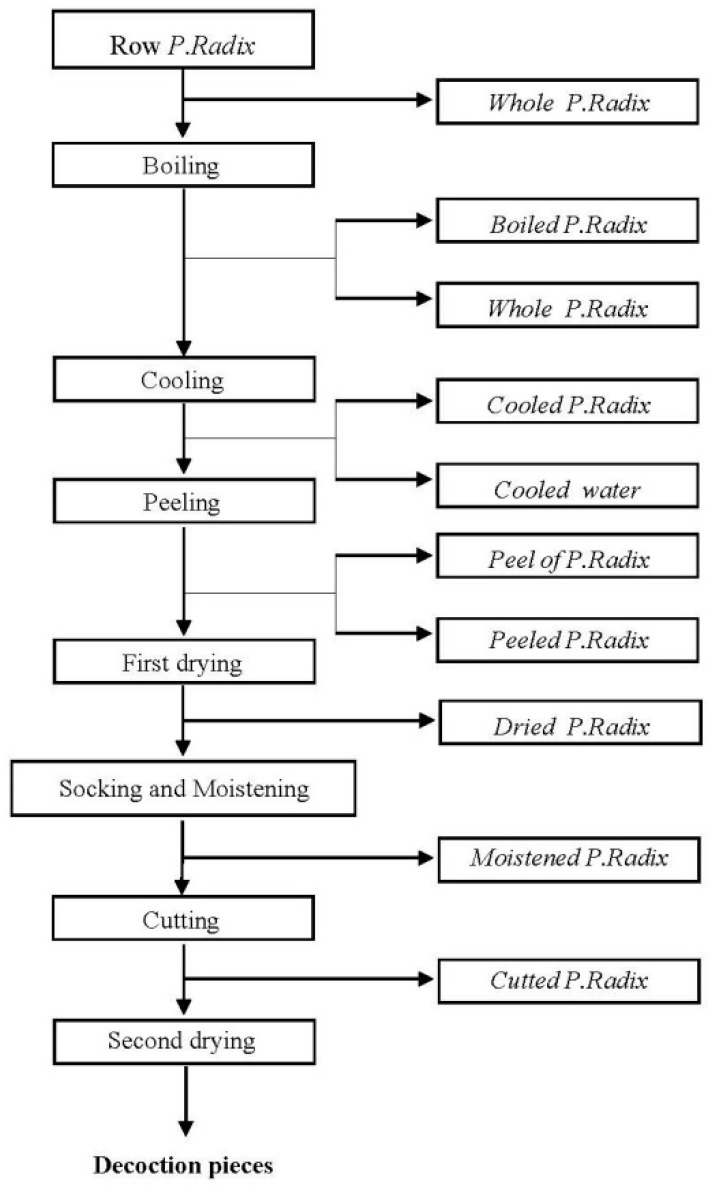
Schematic showing the steps involved in the industrial processing of PRA.

PRA grown in Bozhou city (Anhui, China) without pesticides was selected as the control. The PRA samples were soaked in aqueous solutions containing the three experimental pesticides [15]. To ensure the original deposition of low concentration is enough and to compare the differences of pesticide residues in different concentrations, PRA was soaked in two different pesticide concentrations (two and five times the concentration recommended by local farmers). After soaking for 24 h, the PRA was filtered from the pesticide solutions and dried in the shade. The PRA was then subjected to the following processing steps: boiling, cooling, peeling, drying, moistening, cutting, slicing, and second drying (Figure 1). The pesticide residues were analyzed at each processing step (in triplicate). All samples were stored at −20 °C before analysis.

### 2.3. Extraction and Clean-Up

The pesticide residues were extracted from PRA according to QuEChERS method [14]. Briefly, 2 g of thoroughly homogenized PRA was added to a 50-mL polypropylene centrifuge tube containing 20 mL acetonitrile with 1% acetic acid, 4 g anhydrous magnesium sulfate, and 1 g sodium acetate. The tubes were sealed and shaken vigorously for 1 min by hand followed by centrifugation at 4000 ×g for 5 min. Purification was carried out using dispersive solid-phase extraction as follows: (i) 5 mL of extract was transferred into a polypropylene centrifuge tube containing 60 mg anhydrous magnesium sulfate, 20 mg PSA, and 20 mg C18; (ii) the extract was mixed with sorbent/desiccant for 30 s; (iii) the mixture was centrifuged at 4000 ×g for 5 min; (iv) 2 mL of the mixture was transferred into a 4-mL centrifuge tube and dried by nitrogen on a water bath at 40 °C; and (v) dissolution with 2 mL methanol. The methanol solution was then filtered through a 0.22-μm syringe filter for detection.

### 2.4. UPLC-MS/MS Analysis

UPLC-MS/MS analysis was carried out using a Waters Acquity UPLC instrument interfaced with a XEVO triple quadrupole mass spectrometry system (Waters Co., Milford, MA, USA). The column (2.10 × 100 mm) was packed with 1.7-μm particles (Acquity UPLC BEH C18 column; Waters) was maintained at 40 °C. The mobile phase was 2% methanol in water containing 0.05% formic acid (A) and methanol containing 0.05% formic acid in water (B). The UPLC-MS/MS elution conditions are shown in Table 1. Detection was performed in multiple reaction monitoring (MRM) mode using positive electrospray ionization (3.0 kV). The instrument parameters were optimized to improve sensitivity. The source temperature, cone voltage, desolvation gas flow, cone gas flow, and desolvation temperature were 150 °C, 30 V, 900 L/h, 50 L/h, and 500 °C, respectively. The MRM conditions for the UPLC-MS/MS analyses of the three pesticide residues are shown in Table 2.

### 2.5. Statistical Analysis

Data are expressed as the mean ± standard deviation (SD). Statistically significant mean values (*p* < 0.05 and *p* < 0.01) were calculated using one-way analysis of variance followed by Tukey’s test [16], and an interaction term as parameters in SPSS software (ver. 22.0, SPSS Company, Chicago, USA). Figures were created using Graphad Prism 7 (GraphPad Software, Inc., USA). The limit of detection (LOD) and the limit of quantification (LOQ) were calculated according to the guidelines given in the European Reference Laboratory (EURL) experts’ report. The PFs were calculated as follows based on the Joint FAO/WHO Meeting on Pesticide Residues [15,17]:

$$\mathrm{PF}=\frac{\text{The residue levels of processed commodities}}{\text{The residue levels of the raw commodities}}.$$

A PF value less than one is referred to as a reduction factor and indicates a decrease in pesticide residue during processing, whereas a PF value greater than one is known as a concentration factor and indicates an increase in pesticide concentration during processing [11].

## 3. Results and Discussion

### 3.1. Method Validation

The fortified recovery, precision, LOD, and LOQ values were evaluated for the analysis of the three pesticides residues in PRA. The mean recovery values ranged from 74.2% to 118.2% with a relative standard deviation (RSD) of <9.70% in the fortification range of 0.005 to 0.2 mg/kg. Quantification was performed via the construction of calibration curves based on the peak area of the most intense transition of each pesticide. The calibration plots were linear, with regression coefficients exceeding 0.99. In addition, no interfering peaks were observed in the control samples (Supplementary file Figure S1). The LOQ and LOD of the method were calculated according to the guidelines given in the EURL experts’ report (Table 3).

### 3.2. Effects of Chinese Medicine Processing on Pesticide Residues

The concentrations and characteristics of pesticide residues in food are altered during processing. Numerous studies have shown that food processing can contribute to pesticide dissipation [9,18,19]. However, few studies have been reported on the changes in pesticide residues during CHM processing. In this study, to obtain useful data, the processing conditions corresponded as closely as possible to the processing conditions commonly used in household and industrial practices [20]. Thus, the processing technique used in this study mirrored the processing of PRA in Chinese medicine and included boiling, cooling, peeling, primary drying, moistening, cutting, and secondary drying. The changes in the concentrations of azoxystrobin, difenoconazole, and epoxiconazole, and the associated PFs were examined during each processing step.

#### 3.2.1. Effects of Boiling

**Figure 2:**
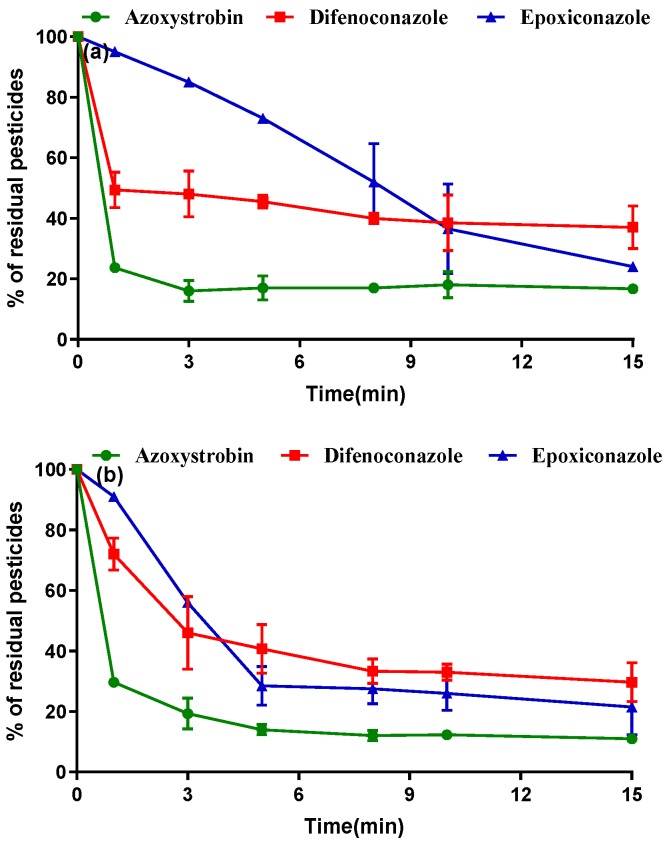
Effects of boiling on the residue levels of three pesticides in *Paeoniae Radix* Alba (PRA) after soaking at two pesticide concentrations: two times (**a**) and five times (**b**) the recommended dosage.

In the commercial processing of PRA, boiling is typically carried out for 5 to 10 min. Thus, 1 to 15 min was selected as the boiling time in this study. As shown in Figure 2, boiling reduced the pesticide concentrations by 61.0% to 89.0%. For both pesticide concentrations (two-fold and five-fold of the typical application concentration), the degradation rates decreased in the following order: azoxystrobin > epoxiconazole > difenoconazole (Figure 2a,b). The degradation rates of the pesticides slowed after 9 min of boiling, after which the pesticide concentrations gradually became stable. The removal percentages of the pesticides exceeded 60% after 15 min. The PRA was soaked in water during boiling; thus, some pesticide residues might have been dissolved in water, and some residues may have decomposed or evaporated under high temperature. Shi et al. [21] reported that the PF of a pesticide in yam was 0.27. Shakoori et al. [8] reported that in rice, the reduction in pesticide residue during boiling was not correlated with the chemical structure or water solubility of the pesticide; instead, they found that the binding strength between the pesticide and the rice matrix along with pesticide volatilization, hydrolysis, and thermal degradation determined the degree of residue removal. These findings are in agreement with our experimental results, which show that boiling is clearly effective for pesticide digestion in PRA.

#### 3.2.2. Effects of Cooling

After boiling, PRA is commonly cooled in water to make the peeling process easier. In this study, cooling did not have an obvious effect on pesticide degradation. During the cooling step, the boiled PRA was simply soaked in cold water, and slightly lower temperatures do not cause pesticide degradation. Other studies have concluded that soaking, particularly in tap water, is not effective for the removal of most pesticides [22]. Most of the pesticides, including those on the surface of the peel and those precipitated from the pulp by osmotic pressure, were removed during the boiling step. Thus, the cooling process had little effect on pesticide residues (Supplementary file Figure S2).

#### 3.2.3. Effects of Peeling

**Figure 3:**
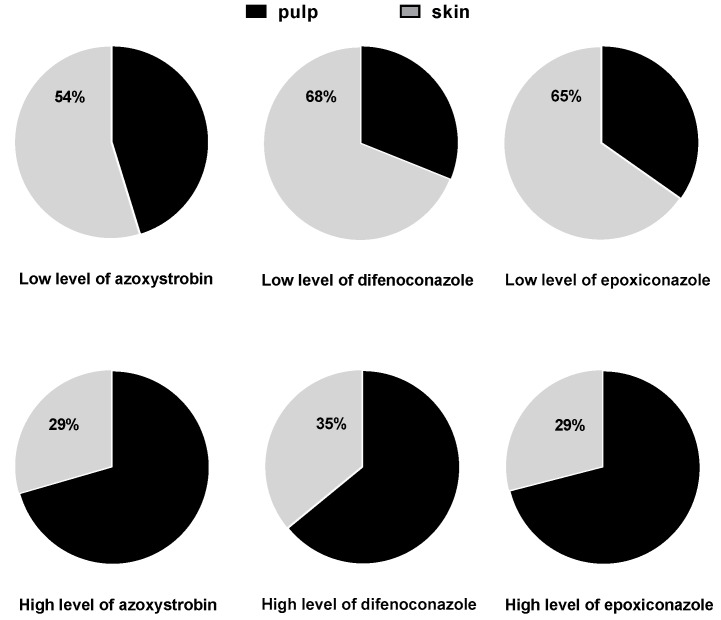
Distributions of the three pesticides in the peel and pulp of PRA treated with low and high pesticide concentrations (two and five times the recommended dosage, respectively).

The PRA skin obtained by peeling was analyzed in this study, along with the pulp (Figure 3). For the samples treated at the lower pesticide concentration, the proportions of pesticide residues were greater in the skin (54% for azoxystrobin, 68% for difenoconazole, and 65% for epoxiconazole) compared to in the pulp. However, at the higher pesticide concentration, the opposite trend was observed (29% for azoxystrobin, 35% for difenoconazole, and 29% for epoxiconazole in the skin). When soaking PRA in pesticide solution, the higher pesticide concentration resulted in more pesticide penetrating into the pulp through the skin; in contrast, the lower pesticide concentration resulted in a lower osmotic pressure, causing more pesticide residue to be found in the skin. This may be because the distribution of pesticide residues varies with different concentrations. Xiao-min Xu et al. [23] reported that in grapes, 90% to 100% of difenoconazole residue and 70% to 95% of azoxystrobin residue was distributed in the skin. For these two pesticides in grapes, only a small quantity migrated into the pulp during planting and storage. Although the skin of PRA is less dense than that of grape, our experimental results suggest that the skin of PRA still prevented some pesticides from migrating into the pulp.

#### 3.2.4. Effects of Soaking and Moistening

**Figure 4:**
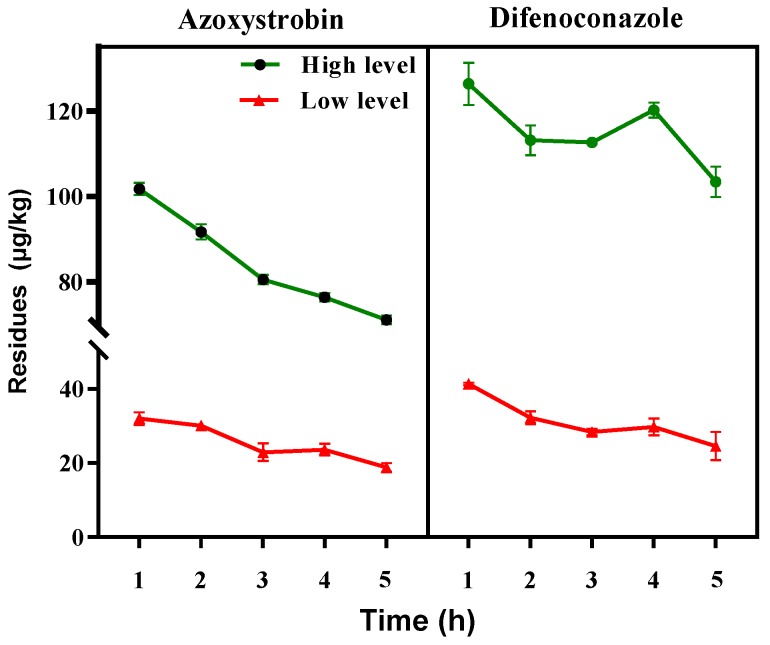
Changes in the concentrations of azoxystrobin and difenoconazole in PRA during soaking and moistening.

After the first drying step, the sample should be soaked, removed from the water, and covered with a damp cloth to allow the water to fully penetrate the PRA sample. This process, which is called moistening, is an essential part of the softening process and facilitates the final slicing the medicinal material. As shown in Figure 4, overall, the pesticide residues in PRA decreased during soaking and moistening, suggesting the pesticide removal depended strongly on time–response effects. After moistening, the residues of azoxystrobin and difenoconazole decreased by 6.92% to 20.14%, respectively. The uniform distribution of moisture in PRA after suffocation may have diluted the pesticide residues.

#### 3.2.5. Effects of Drying

**Figure 5:**
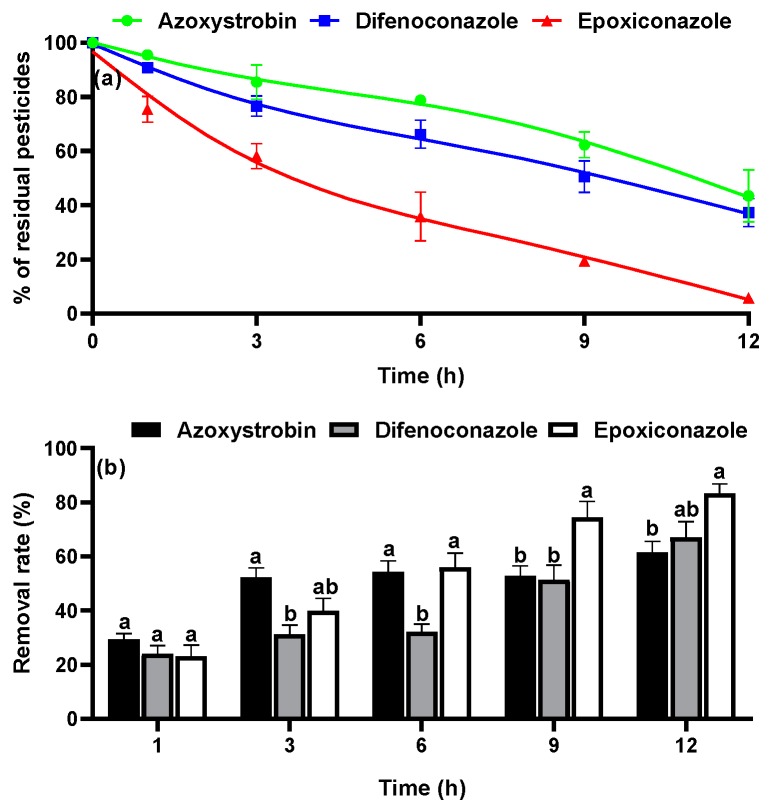
Elimination of the three pesticides during the first drying process. Effects of drying temperatures on the levels of pesticide residues in *P. Radix* at two times the recommended dosage and the removal of the three pesticides at the same temperature (40 °C) for treatment with pesticide concentrations of five times the recommended dosage. Different lowercase letters at the top of columns represent significant differences in residue levels at a *p*-value of 0.05.

PRA was subjected to two drying processes, one after peeling and a second after slicing. The first drying step was carried out at three temperatures: 40 °C, 60 °C, and 80 °C. It is observed that drying has an obvious effect on pesticide dissipation. The trends were first decreased rapidly and then more slowly during the drying processes. Azoxystrobin residue was greatly reduced during the first drying step, particularly at 60 °C, when the residue was reduced by nearly 60% in 3 h (Figure 5a,b). After 12 h of drying, the pesticide residues at different temperatures followed the following order 40 °C > 60 °C > 80 °C. Compared to drying at 60 °C, the pesticide removal rates were slightly lower at 80 °C. This might be because the higher temperature caused the moisture on the surface of PRA to evaporate quickly, which was disadvantageous to pesticide precipitation and degradation. Among the three pesticides, epoxiconazole had the highest degradation rate during the first drying process, while azoxystrobin had the lowest degradation rate.

**Figure 6:**
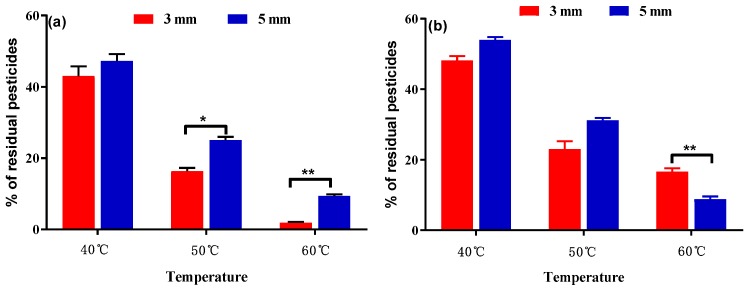
Effects of slice thickness and drying temperature on azoxystrobin (**a**) and difenoconazole (**b**) removal during the second drying step. Bars marked with an asterisk indicate significant differences with respect to the removal rate (* *p*-value < 0.05 and ** *p*-value < 0.01).

In the final steps of PRA processing, the samples were sliced into medicinal slices and dried for a second time for 6 h. This final step was carried out at three temperatures (40 °C, 50 °C, and 60 °C), and two slice sizes were created (3 and 5 mm). The degree of pesticide degradation increased with increasing temperature during the second drying step. Under the same drying temperature, pesticide degradation was greater for the 3-mm slices compared to the 5-mm slices (Figure 6), likely because the thinner slices were more conducive to pesticide elimination. For example, the percent of residual azoxystrobin in 3-mm slices at 60 °C drying was 1.87%, and the corresponding in 5-mm slices was 9.37%, as well as 16.63% and 8.80% of residual pesticide for difenoconazole in 3-mm and 5-mm slices, respectively.

### 3.3. Processing Factors 

Pesticides and fertilizers are used to control diseases and pests during CHM production, ultimately improving the CHM yield and quality. However, the complex processing procedures applied to CHMs can greatly reduce the pesticide residues present in the final products. Hence, PFs are important for assessing the risks of pesticides in CHMs. The calculated PFs for the three pesticides after processing in this study are shown in Table 4. All PFs were less than one, indicating that the residue concentrations decreased during processing. The highest rate of pesticide removal was observed during the boiling step (PF < 0.37 for all three pesticides). The different initial pesticide concentrations during soaking generated different osmotic pressures, which resulted in different pesticide distributions in the pulp and skin of PRA. For the peeling step, the PFs ranged from 0.31 to 0.45 at the high initial pesticide concentration and from 0.64 to 0.71 at the low concentration. Due to inadequate initial deposition, epoxiconazole residues were not detectable after the first drying step. However, based on the first four steps, the PF for epoxiconazole did not exceed 0.02.

## 4. Conclusions

In this study, the changes in the concentrations of three pesticides, azoxystrobin, difenoconazole, and epoxiconazole, were examined during different PRA processing steps (boiling, cooling, drying, peeling, soaking, moistening, and cutting), and the associated PFs were calculated. The PFs of each step were generally less than one, and the PFs of the overall process were lower than 0.027 for all pesticides. Thus, the processing of PRA obviously reduced the concentrations of pesticide residues. The boiling process was the most effective step for pesticide degradation, with PFs in the range of 0.11 to 0.37. The two drying processes also resulted in significant pesticide degradation (PF = 0.32–0.48) resulting from pesticide volatilization, hydrolysis, and thermal degradation. Overall, the results demonstrate that pesticide residues in PRA are significantly reduced by industrial processing. In a follow-up study, we will explore the dietary risks associated with drinking CHM decoctions. The results provide valuable data that may be useful for establishing maximum residue limits in PRA and assessing the amount of pesticide residues in PRA under industrial conditions.

## Figures and Tables

**Table 1:** Elution conditions for UPLC-MS/MS analysis

Time (min)	Flow Rate (mL/min)	A % ^a^	B % ^b^
0	0.4	90	10
0.25	0.4	90	10
7.00	0.4	0	100
8.50	0.4	0	100
8.51	0.4	0	100
10.00	0.4	90	10

^a^ A, 2% methanol in water containing 0.05% formic acid; ^b^ B, methanol containing 0.05% formic acid in water.

**Table 2:** Multiple reaction monitoring (MRM conditions) during the HPLC-MS/MS analyses of three pesticide residues.

Compound	Precursor Ion (*m/z*) ^a^	Product Ions (*m/z*)	Dwell Time (s)	Cone (v)	Collision (v)
Azoxystrobin	404.10	372.05* ^b^	0.008	17	14
		329.00			31
Epoxiconazole	330.05	141.10	0.008	25	21
		121.10*			18
Difenoconazole	406.10	251.00*	0.01	37	25
		337.05			17

^a^*m/z*, mass-to-charge ratio; ^b^ Asterisk (*) represent the quantifier ion

**Table 3:** Validation parameters for the UPLC-MS/MS determination of three pesticides in processed samples.

Matrixes	Pesticides	Linearity-Correlation Coefficient	Limit of Detection (LOD) (μg/kg)	Limit of Quantification (LOQ) (μg/kg)	Recovery ± RSD (%) (*n* = 5)		
					Level I	Level II	Level III
					(5.00 μg/kg)	(50.00 μg/kg)	(200 μg/kg)
Raw PRA.	Azoxystrobin	0.9977	0.33	1.05	79.50 ± 3.42	102.59 ± 3.44	118.23 ± 3.27
	Difenoconazole	0.9986	0.09	0.29	106.52 ± 3.72	91.90 ± 7.34	93.60 ± 5.76
	Epoxiconazole	0.9987	0.71	2.41	109.30 ± 7.21	105.80 ± 4.56	92.37 ± 4.28
Peels	Azoxystrobin	0.9989	1.12	3.51	97.50 ± 3.05	87.70 ± 8.17	84.69 ± 5.50
	Difenoconazole	0.9987	0.13	0.37	84.73 ± 2.94	84.30 ± 4.39	95.14 ± 4.75
	Epoxiconazole	0.9974	1.4	4.08	95.33 ± 7.59	109.15 ± 6.37	75.39 ± 4.66
Water	Azoxystrobin	0.9997	0.5	1.83	78.00 ± 6.91	78.40 ± 1.36	76.60 ± 1.24
	Difenoconazole	0.9965	0.06	0.18	118.20 ± 2.45	79.50 ± 6.17	74.20 ± 9.70
	Epoxiconazole	0.9991	0.92	2.93	99.15 ± 3.14	109.78 ± 6.34	112.16 ± 7.84

**Table 4:** Processing factors (PFs) for the three pesticides after different processes (*n* = 3).

Process	Azoxystrobin		Difenoconazole		Epoxiconazole	
	2×	5×	2×	5×	2×	5×
Boiling	0.17	0.11	0.37	0.30	0.24	0.14
Cooling	0.85	0.94	0.82	0.96	0.90	0.89
Peeling	0.45	0.70	0.31	0.64	0.34	0.71
First drying	0.45	0.43	0.33	0.37	0.17	0.06
Soaking and moistening	0.58	0.70	0.63	0.84	<LOQ	<LOQ
Second drying	0.40	0.43	0.32	0.48	<LOQ	<LOQ
Overall process	0.006	0.009	0.006	0.027	<LOQ	<LOQ

## Supplementary Materials

The following are available online https://www.mdpi.com/1660-4601/16/12/2196/s1, Figure S1. Typical chromatograms of pesticides in control (a) and spiked PRA sample (b) with a standard mixture at 50 μg/kg; Figure S2. Residues of epoxiconazole, difenoconazole, and azoxystrobin in PRA during cooling.
