# Supplementary material for: Dissipation Behavior of Three Fungicides during the Industrial Processing of Paeoniae Radix Alba and Associated Processing Factors
Source: Int J Environ Res Public Health. 2019 Jun 21;16(12):2196. doi: 10.3390/ijerph16122196 (PMC6617218; doi:10.3390/ijerph16122196)
Supplement: Supplementary file 1 [file ijerph-16-02196-s001.pdf]

Dissipation Behavior of Three Fungicides during the Industrial Processing of *Paeoniae Radix* Alba and Associated Processing Factors

Sheng-Nan Li [1,†], Ming-Na Sun [3,†], Fan Wang [1], Xing Xu [2], Xin-Hong Zhang [1], Jin-Juan Ma [1], Jin-Jing Xiao [1,2], Min Liao [1,2] and Hai-Qun Cao [2,3*]

1 School of Plant Protection, Anhui Agricultural University, Hefei 230036, China; 13210615585@163.com (S.-N.L.); xiaojj187012@163.com (J.-J.X.); liaomin3119@126.com (M.L.); fxhlrh@163.com(F.W.); majinjuan1024@163.com(J.-J.M.); zxh1060559211@163.com(X.-H.Z.)
2 Provincial Key Laboratory for Agri-Food Safety, Anhui Agricultural University, Hefei 230036, China; haiquncao@163.com (H.-Q.C.); xu99xing@163.com (X.X.)
3 Institute of Plant Protection and Agro-Product Safety, Anhui Academy of Agricultural Sciences, Key Laboratory of Agro-Product Safety Risk Evaluation (Hefei), Ministry of Agriculture, Hefei 230031, China; sunmingna@126.com (M.-N.S).
* Correspondence: haiquncao@163.com; Tel.: +86-0551-6578-5730; Fax: +86-0551-6578-5730
† These authors contributed equally to this work.

Received: date; Accepted: date; Published: date

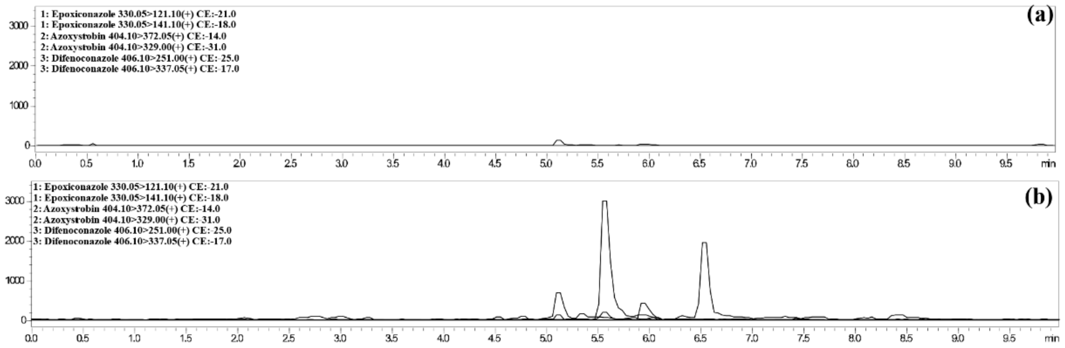

Figure S1. Typical chromatograms of pesticides in control (a) and spiked sample (b) with a standard mixture at 50 μg/kg.

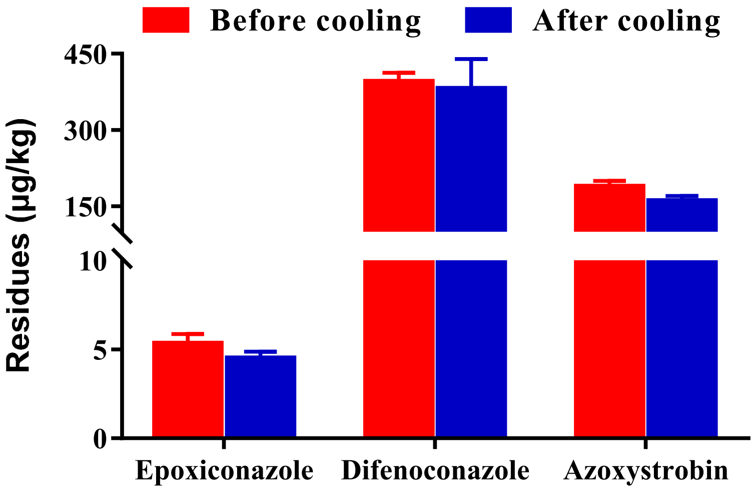

Figure S2. Residues of epoxiconazole, difenoconazole, and azoxystrobin in PRA during cooling
